# Nanoarchitectonics for advanced applications in energy, environment and biology: Method for everything in materials science

**DOI:** 10.3762/bjnano.14.60

**Published:** 2023-06-19

**Authors:** Katsuhiko Ariga

**Affiliations:** 1 Research Center for Materials Nanoarchitectonics, National Institute for Materials Science, 1-1 Namiki, Tsukuba 305-0044, Japan; 2 Department of Advanced Materials Science, Graduate School of Frontier Sciences, The University of Tokyo, 5-1-5 Kashiwanoha, Kashiwa 277-8561, Japan

**Keywords:** functional materials, materials science, nanoarchitectonics, nanotechnology

The advancement of society depends on functional materials that are available and systems that are generated by the population. We can say that the development of advanced civilizations happens in parallel and is due to the development of functional materials. In the beginning of the civilization, humans used to extract materials directly from nature and process them for personal use. Later, we learned how to create materials not found in nature through various chemical reactions. Such material creation quickly progressed because of the introduction and development of various scientific fields, which mainly developed in the last century. Organic chemistry, inorganic chemistry, polymer chemistry, supramolecular chemistry, coordination chemistry, and various materials science fields have enabled the creation of functional materials that improved human life. In the course of their development, we have learned that the function of a material depends not only on the material itself but also on the precision of its internal structure. This is where nanotechnology has brought about a revolution. Nanotechnology has enabled the observation and analysis of the properties of objects at the nanoscale level, down to molecules and atoms. Then, by understanding and creating new nanostructures it became possible to create materials with unprecedented high functionality. Society has brought about amazing progress in the creation of new materials by extracting and processing natural resources, and using these resources in nanofabrication.

**Figure 1:**
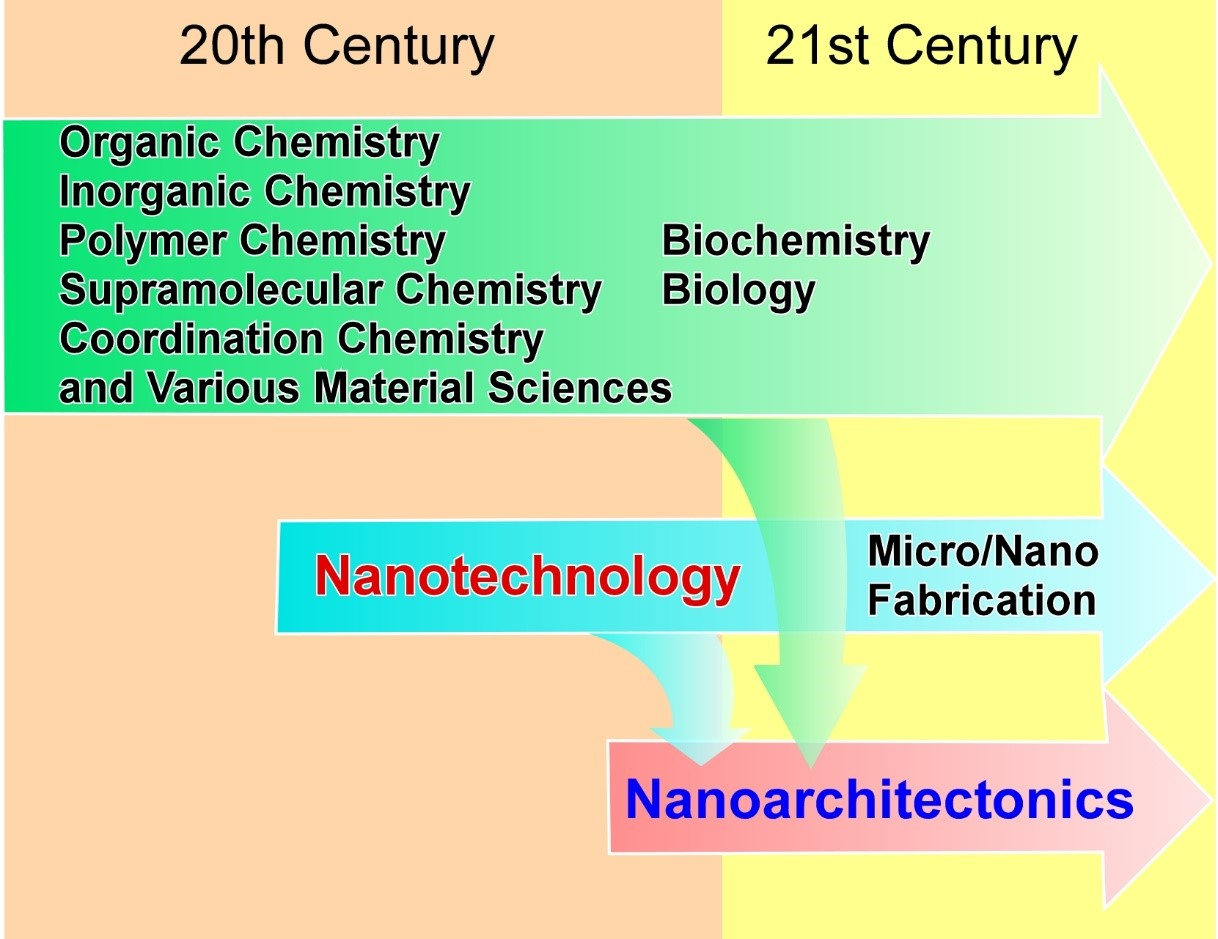
Historical flow to bridge nanotechnology amd materials science.

In order to establish a methodology to generate new materials which takes advantage of the properties of nanostructures, it is necessary to integrate science and technology at the nanoscale level while including various materials chemistry fields, as initiated by nanotechnology. This concept is defined as nanoarchitectonics (Figure 1) [1–2]. Nanoarchitectonics can also be considered a post-nanotechnology concept [3]. Nanotechnology was proposed by Richard Feynman in the 20th century [4–5] whereas nanoarchitectonics was proposed by Masakazu Aono in the early 21st century [6]. Nanoarchitectonics is a methodology for architecting functional material systems from components at the nanoscale (i.e., atoms, molecules, and nanomaterials) following the footsteps of nanotechnology, which pioneered the science at that length scale. Such methodologies were also touched upon in the bottom-up fabrication of materials using supramolecular chemistry and other methods [7–8]. Nanoarchitectonics encompasses these methods and integrates them into a broader field of research. Therefore, rather than creating completely new concepts, nanoarchitectonics works to universally integrate disciplines. Nanoarchitectonics is the integration of nanotechnology, not only in the fields of chemistry and materials science, such as the creation of materials as described above, but also in micro- and nanofabrication sectors as well as in bio-related sciences [9]. Nanoarchitectonics bridges the worlds of nanotechnology and materials science integrating all related scientific fields in between.

Fundamentally, all materials are composed of atoms and molecules, so nanoarchitectonics is applicable to all materials. As an equivalent to the theory of everything in physics [10], nanoarchitectonics can be considered as a method for everything in materials science [11]. Nanoarchitectonics, which is applicable to many materials systems, is also relevant to many other fields. By selecting only papers that include the word “nanoarchitectonics” in the title, one would appreciate that these manuscripts are not only coming from basic fields of research such as material fabrication [12–13], structural control [14–15], elucidation of physical phenomena [16–17], and basic bio-related sciences [18–19], but also from applied fields such as catalysis [20–21], sensors [22–23], devices [24–25], environmental research [26–27], energy [28–29], and biomedical [30–31] fields. In this thematic issue entitled “Nanoarchitectonics for advanced applications in energy, environment and biology”, the authors also discuss coordination-assembled myricetin nanoarchitectonics [32], nanoarchitectonics for membranes with enhanced gas separation capabilities [33], nanoarchitectonics of the cathode of Li–O_2_ batteries [34], nanoarchitectonics in moist-electric generation [35], nanoarchitectonics for drug delivery systems [36], nanoarchitectonics to entrap living cells [37], among other papers in which the concept of nanoarchitectonics has been applied to a variety of targets.

Nanoarchitectonics integrates many existing disciplines and bridges nanotechnology and materials science. Due to the universality of the concept, it can be regarded as method for everything in materials science as shown in the manuscripts published in this thematic issue. By using nanoarchitectonics one can create new functional materials, help with societal development, and solve various problems, such as environmental issues, for instance.

Katsuhiko Ariga

Tsukuba, June 2023
